# O-GlcNAcylation in tumorigenesis and its implications for cancer therapy

**DOI:** 10.1016/j.jbc.2024.107709

**Published:** 2024-08-22

**Authors:** Dize Zhang, Yihang Qi, Hiroyuki Inuzuka, Jing Liu, Wenyi Wei

**Affiliations:** 1 Department of Urology, The First Affiliated Hospital of Xi'an Jiaotong University, Xi'an, China; 2 Key Laboratory for Tumor Precision Medicine of Shaanxi Province, The First Affiliated Hospital of Xi’an Jiaotong University, Xi’an, China; 3 Department of Pathology, Beth Israel Deaconess Medical Center, Harvard Medical School, Boston, Massachusetts, United States

**Keywords:** O-GlcNAcylation, tumorigenesis, cancer therapy, chemical-induced proximity

## Abstract

O-linked N-acetylglucosaminylation (O-GlcNAcylation) is a dynamic and reversible posttranslational modification that targets serine and threonine residues in a variety of proteins. Uridine diphospho-N-acetylglucosamine, which is synthesized from glucose *via* the hexosamine biosynthesis pathway, is the major donor of this modification. O-GlcNAc transferase is the sole enzyme that transfers GlcNAc onto protein substrates, while O-GlcNAcase is responsible for removing this modification. O-GlcNAcylation plays an important role in tumorigenesis and progression through the modification of specific protein substrates. In this review, we discuss the tumor-related biological functions of O-GlcNAcylation and summarize the recent progress in the development of pharmaceutical options to manipulate the O-GlcNAcylation of specific proteins as potential anticancer therapies.

O-GlcNAcylation is a type of posttranslational protein modification (PTM) that involves the addition of N-acetylglucosamine (GlcNAc) to the hydroxyl group of serine/threonine residues on target proteins (1). This process is dynamic and reversible and is controlled by two well-studied enzymes, O-GlcNAc transferase (OGT) and O-GlcNAcase (OGA, Fig. 1). OGT is responsible for transferring GlcNAc from Uridine diphosphate-N-acetylglucosamine (UDP-GlcNAc) to specific proteins for O-linked glycosylation (2), whereas OGA is responsible for removing this modification (3). The 14-3-3 proteins have been reported to be reader proteins of O-GlcNAcylation (4). UDP-GlcNAc, produced by the hexosamine biosynthesis pathway, is the main donor of O-GlcNAcylation, which can be influenced by a variety of upstream factors, such as glucose metabolism, insulin signaling, and the cellular stress response (5). O-GlcNAcylation plays a pivotal role in regulating a plethora of cellular functions, largely through the modification of specific substrate proteins, including transcription factors/cofactors (6), epigenetic regulators (7), protein kinases/phosphatases (8, 9, 10), and E3 ubiquitin ligases (11). Cancer cells usually have high OGT levels but low OGA expression levels, and elevated O-GlcNAcylation appears to be a general characteristic of cancer cells (12, 13, 14). To date, O-GlcNAcylation has been identified in thousands of proteins *via* low-throughput and high-throughput methods, as reviewed recently by others (5, 11, 15). Mechanistically, O-GlcNAcylation can occur on serine/threonine residues, which are critical phosphorylation sites, thus disrupting the phosphorylation-mediated regulation of protein substrates (16). In addition, O-GlcNAcylation might also protect proteins from ubiquitination-mediated protein degradation (17), providing another layer of regulation over protein turnover (Fig. 2).

**Figure 1:**
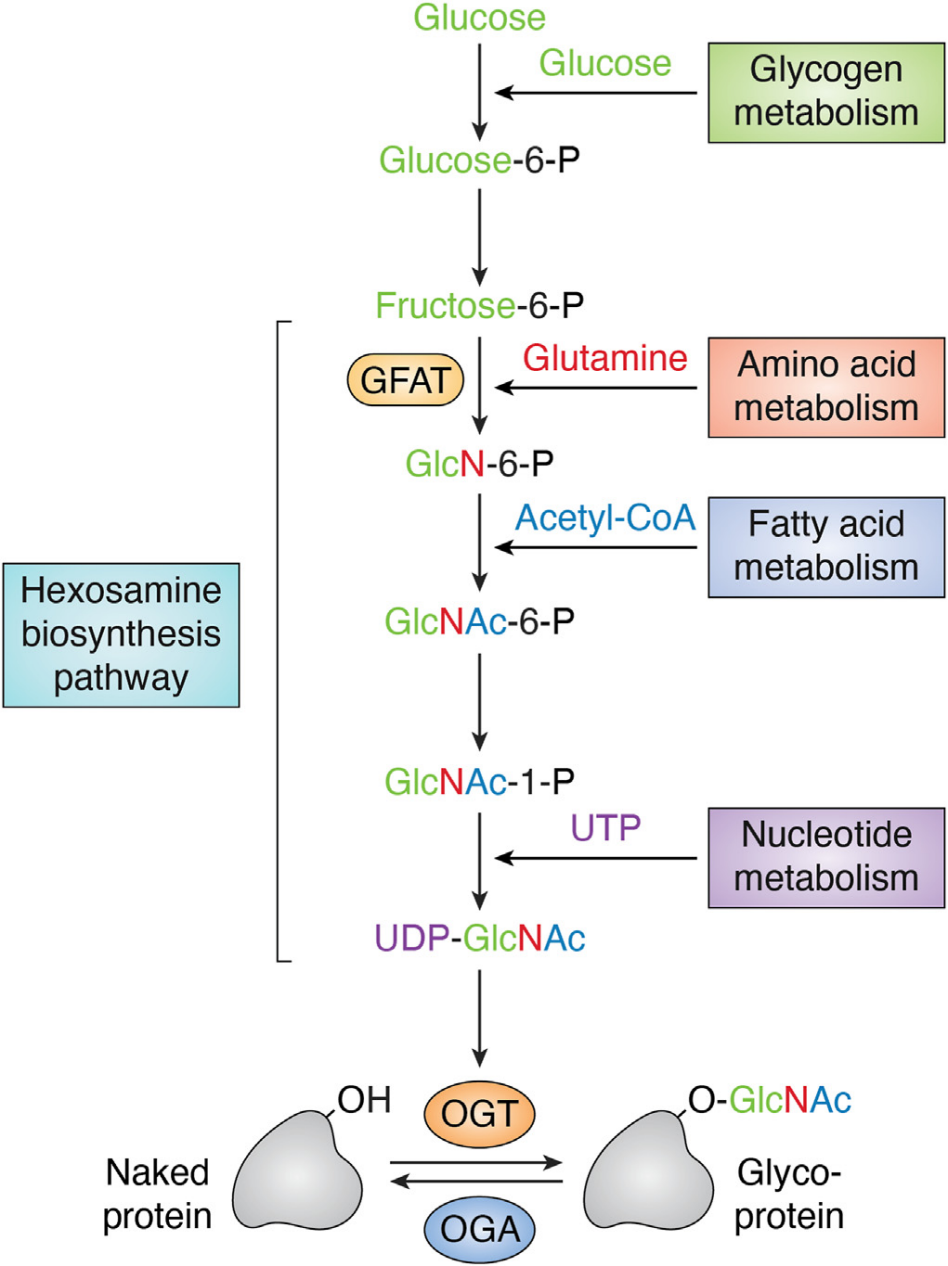
**O-GlcNAcylation integrates multiple types of cellular metabolic signaling pathways into protein modifications.** Glc: glucosamine, Glc-6-P: glucose-6-phosphate, Fruc-6P: fructose-6-phosphate, GlcN-6-P: glucosamine-6-phosphate, Acetyl-CoA: acetyl coenzyme A, GlcNAc-6-P: N-acetyl-D-glucosamine 6-phosphate, GlcNAc-1-P: N-acetyl-D-glucosamine 1-phosphate, UTP: uridine 5′-triphosphate, UDP-GlcNAc: uridine diphosphate N-acetylglucosamine OGT: O-GlcNAc transferase, OGA: O-GlcNAcase. O-GlcNAcylation, O-linked N-acetylglucosaminylation.

**Figure 2:**
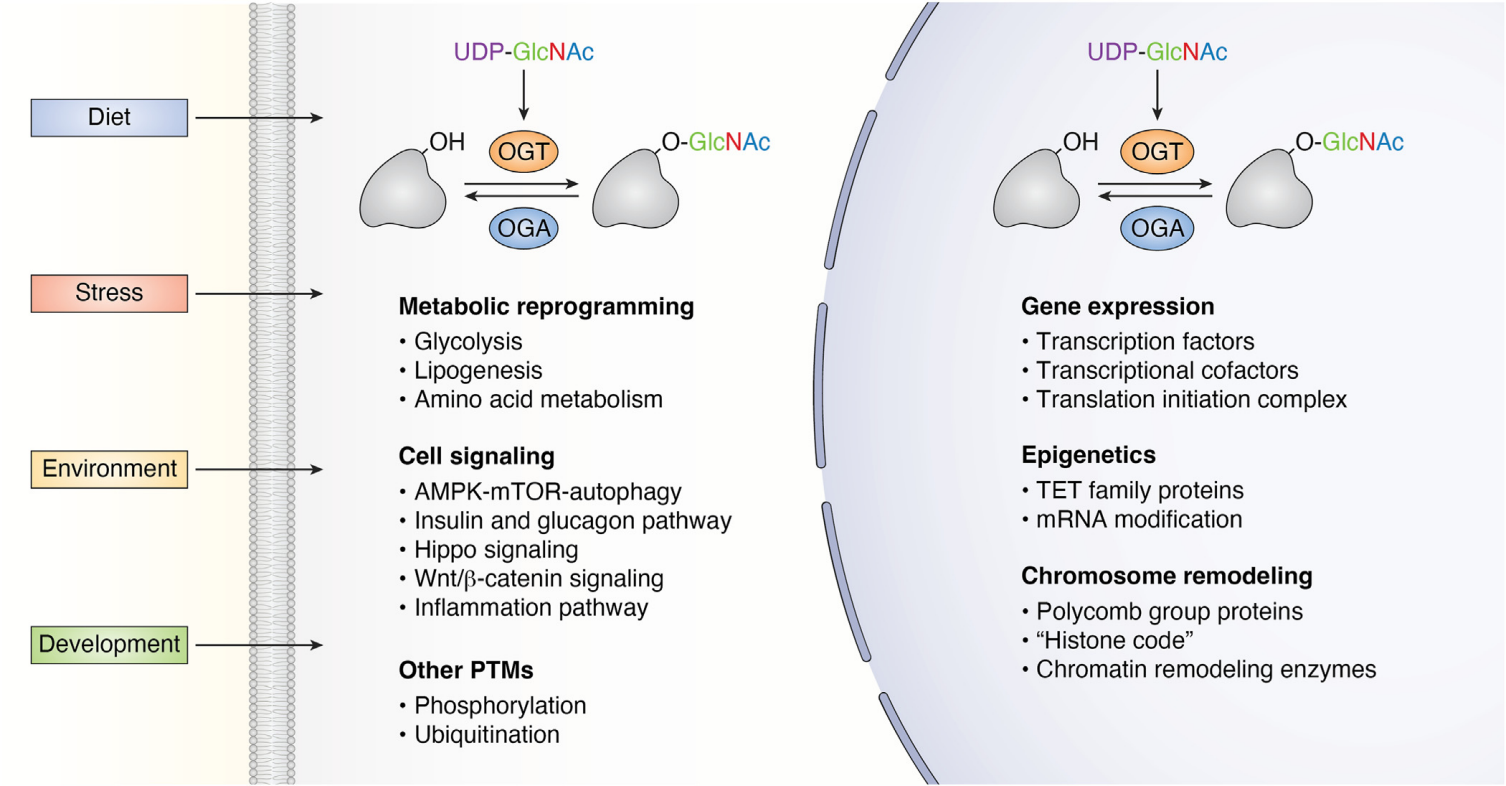
**Upstream biological cues and downstream effects of protein O-GlcNAcylation**. O-GlcNAcylation functions as a pivotal sensor for extracellular and intracellular cues and transduces these inputs onto diverse cell signaling pathways in part through the modification of different protein substrates, thus remodeling cellular functions, such as cell metabolism and cell signaling in the cytoplasm, as well as transcriptional regulation and chromosome remodeling in the nucleus. O-GlcNAcylation, O-linked N-acetylglucosaminylation.

Many efforts have been made to develop therapeutic options for cancer through the manipulation of the O-GlcNAcylation of proteins, primarily using specific inhibitors of OGT or OGA (15, 18). Recently, chemical-induced proximity (CIP) methods have been adopted to manipulate the PTMs of proteins, including ubiquitination, phosphorylation, acetylation, and O-GlcNAcylation, suggesting that this method is a powerful tool and potential therapeutic approach (19, 20). Although none of these CIPs has been approved by the Food and Drug Administration for clinical use, several proteolysis targeting chimeras (PROTACs) and molecular glue degraders have shown promising outcomes in early clinical trials (21, 22). Based on the current research progress on O-GlcNAcylation, in this review, we summarize the underlying mechanisms of O-GlcNAcylation in human cancer and summarize the current induction methods and mouse models of O-GlcNAcylation relevant to *in vivo* experiments. In addition, the achievements of biomedical engineering editing technology in the development of inhibitors that target O-GlcNAcylation are listed.

## O-GlcNAcylation regulates cancer cell metabolism

Cells respond to extracellular and intracellular cues through diverse signaling pathways, and those signals are usually dysregulated in cancer cells. O-GlcNAcylation is sensitive to nutrients, hormones, and stress, thus enabling cells to reshape their metabolism and fate to adapt to these environmental inputs. Given that glucose is the major source of UDP-GlcNAc production, the disruption of glucose uptake and metabolism affects global intracellular O-GlcNAcylation levels. For example, glucose starvation increases the O-GlcNAcylation of leucyl-tRNA synthetase 1 (LARS1) at Ser-1042, which reduces the affinity of LARS1 for leucine by promoting unc-51-like kinase 1 (ULK1)-mediated LARS1 phosphorylation at the leucine-binding site, thus integrating glucose and amino acid metabolism (23). Compared with normal cells, cancer cells have a different metabolic homeostasis and prefer glycolysis rather than oxidative phosphorylation, termed the Warburg effect, and the O-GlcNAcylation of proteins is also not the same as that in normal cells.

**Figure 3:**
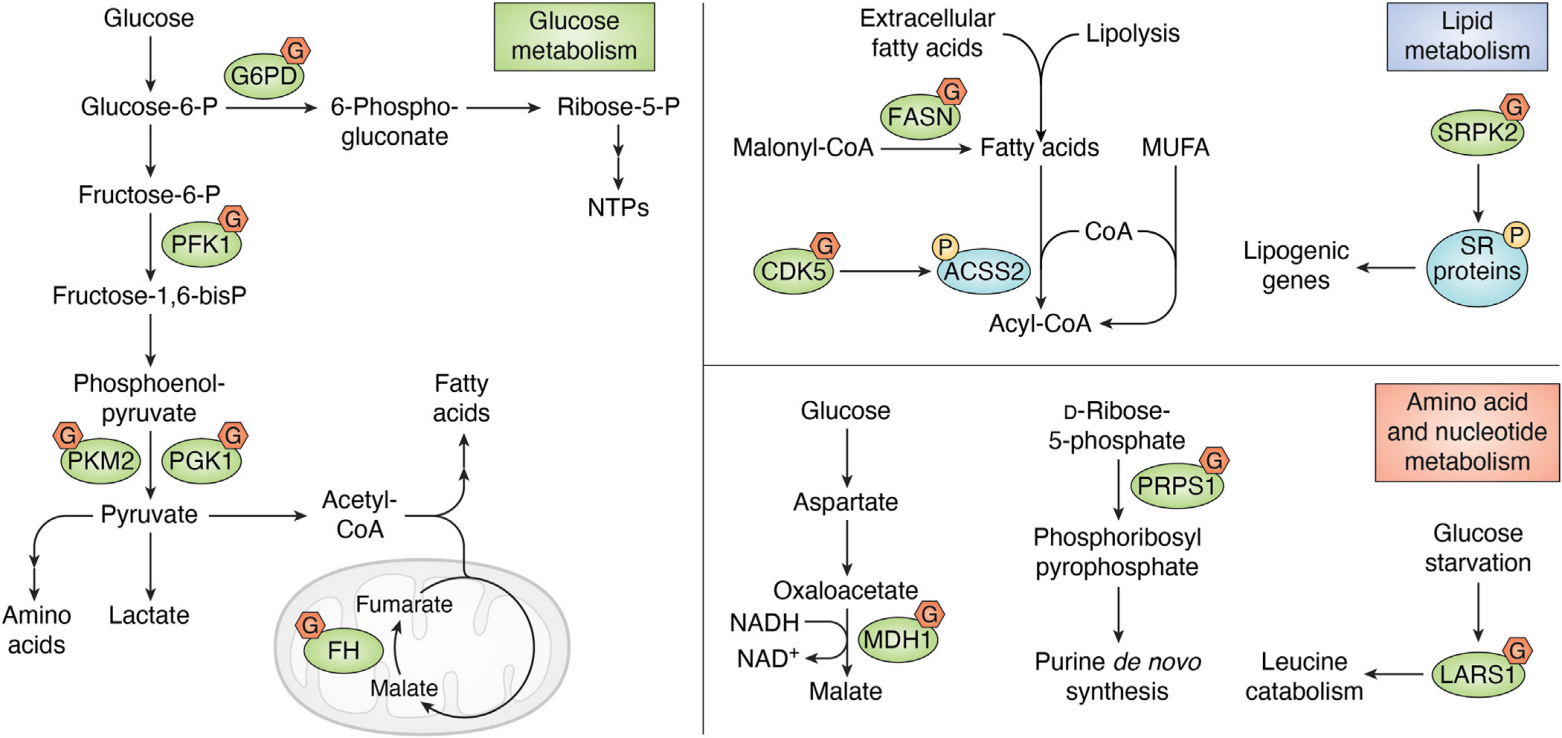
**O-GlcNAcylation regulates cancer cell metabolism**. O-GlcNAcylation occurs on multiple enzymes involved in the metabolism of glucose, lipids, and amino acids, thus reshaping cellular metabolism to promote cancer. G: O-GlcNAcylation. P: phosphorylation. O-GlcNAcylation, O-linked N-acetylglucosaminylation.

O-GlcNAcylation has a dominant effect on cancer metabolism; for example, OGT inhibition reduces glucose uptake and lactate production, thus suppressing the proliferation of prostate cancer cells (24). Many enzymes involved in glycolysis and the pentose phosphate pathway (PPP) have been reported to undergo O-GlcNAcylation, serving as another layer regulating cancer metabolism (Fig. 3). Phosphofructokinase 1 (PFK1) is the first enzyme that commits glucose to glycolysis and catalyzes the irreversible conversion of fructose-6-phosphate to fructose-1,6-bisphosphate. O-GlcNAcylation of Ser-529 of PFK1 in response to hypoxia inhibits its enzymatic activity and rewires cancer cell metabolism to the PPP, which represents a potential therapeutic option to target cancer metabolism (25). Glucose-6-phosphate dehydrogenase (G6PD) is the rate-limiting enzyme of the PPP, and hypoxia triggers its O-GlcNAcylation at Ser-84 in human lung cancers, leading to G6PD activation and metabolic flux into the PPP to produce intermediates of nucleotide and lipid biosynthesis in cancer cells (26). Phosphoglycerate kinase 1 (PGK1), a glycolytic enzyme that reversibly converts 1,3-diphosphoglycerate to 3-phosphoglycerate, can also be GlcNAcylated (27). Mechanistically, the O-GlcNAcylation of PGK1 at Thr-255 not only increases PGK1 kinase activity to increase lactate production but also triggers the translocation of PGK1 into mitochondria to phosphorylate and inhibit pyruvate dehydrogenase in colon cancer cells. Furthermore, blocking O-GlcNAcylation *via* the PGK1-T255V mutation inhibits colon cancer cell proliferation *in vitro* and retards tumor growth *in vivo* (27). Pyruvate kinase M2 (PKM2), an isoform specifically expressed in cancer cells, serves as a key driver of the Warburg effect. The O-GlcNAcylation levels of PKM2 at Thr-405 and Ser-406 are increased in both cancer cells and patient tumors (28), which leads to the destabilization of the active PKM2 tetramer and the nuclear translocation of PKM2 to enhance the Warburg effect, while blocking this O-GlcNAcylation attenuates tumor growth *in vivo* (28). Moreover, GlcNAcylation can be antagonized by ULK1-mediated PKM2 phosphorylation at Ser-333 (29). In addition to the abovementioned examples, many other metabolic enzymes in the tricarboxylic acid cycle are also substrates of O-GlcNAcylation, among which fumarate hydratase (FH) is O-GlcNAcylated at the Ser-75 residue, the same site as AMPK-mediated phosphorylation, and the O-GlcNAcylation of this site maintains tumor growth under glucose deficiency in pancreatic cancer patients (30).

Lipid metabolism has attracted increasing attention in recent decades, and recent studies have revealed that several enzymes involved in lipid metabolism, such as fatty acid synthase (FASN), are O-GlcNAcylated. FASN is the sole enzyme for endogenous long-chain fatty acid synthesis and the rate-limiting enzyme of *de novo* lipogenesis. O-GlcNAcylation of FASN enhances its activity, thus protecting cancer cells from starvation, and dual inhibition of O-GlcNAcylation and FASN synergistically induces cancer cell death (31). In addition to directly modifying metabolic enzymes, O-GlcNAcylation can also regulate cellular metabolism through transcriptional, posttranscriptional, and posttranslational mechanisms. For example, serine/arginine-rich protein kinase 2 (SRPK2), a kinase for pre-mRNA splicing factors, can be O-GlcNAcylated at its nuclear localization signal to facilitate its import into the nucleus to phosphorylate pre-mRNA splicing factors, thus promoting the pre-mRNA splicing of lipogenic genes (32). Moreover, OGT regulates lipogenesis by indirectly increasing cyclin-dependent kinase 5-mediated Ser-267 phosphorylation of acetyl-CoA synthetase 2 (ACSS2) in glioblastoma cells (33).

Recent studies focusing on cancer metabolism have revealed a tumor type-specific metabolic vulnerability, such as glutamine and methionine metabolism (34). Pancreatic ductal adenocarcinomas (PDACs) rely on glutamine to sustain proliferation, and malate dehydrogenase 1 (MDH1), a key enzyme in glutamine metabolism, is highly O-GlcNAcylated at Ser-189 in PDAC patients, which enhances MDH1 enzymatic activity and glutamine metabolism, thus providing an Achilles heel for the treatment of PDAC (35). Furthermore, O-GlcNAcylation also functions in regulating nucleotide metabolism by modifying phosphoribosyl pyrophosphate synthetase 1 (PRPS1), a key enzyme in *de novo* nucleotide synthesis, at the Ser-83 and Thr-166 sites. O-GlcNAcylation of PRPS1 triggers its hexamerization and inhibits AMPK-mediated PRPS1 phosphorylation, thus increasing its activity and promoting tumorigenesis in lung cancer (36). Notably, OGT-mediated O-GlcNAcylation promotes glucose metabolism in cancer, indicating the potential of OGT inhibitors to serve as novel anticancer treatments.

## O-GlcNAcylation regulates cancer cell signaling

**Figure 4:**
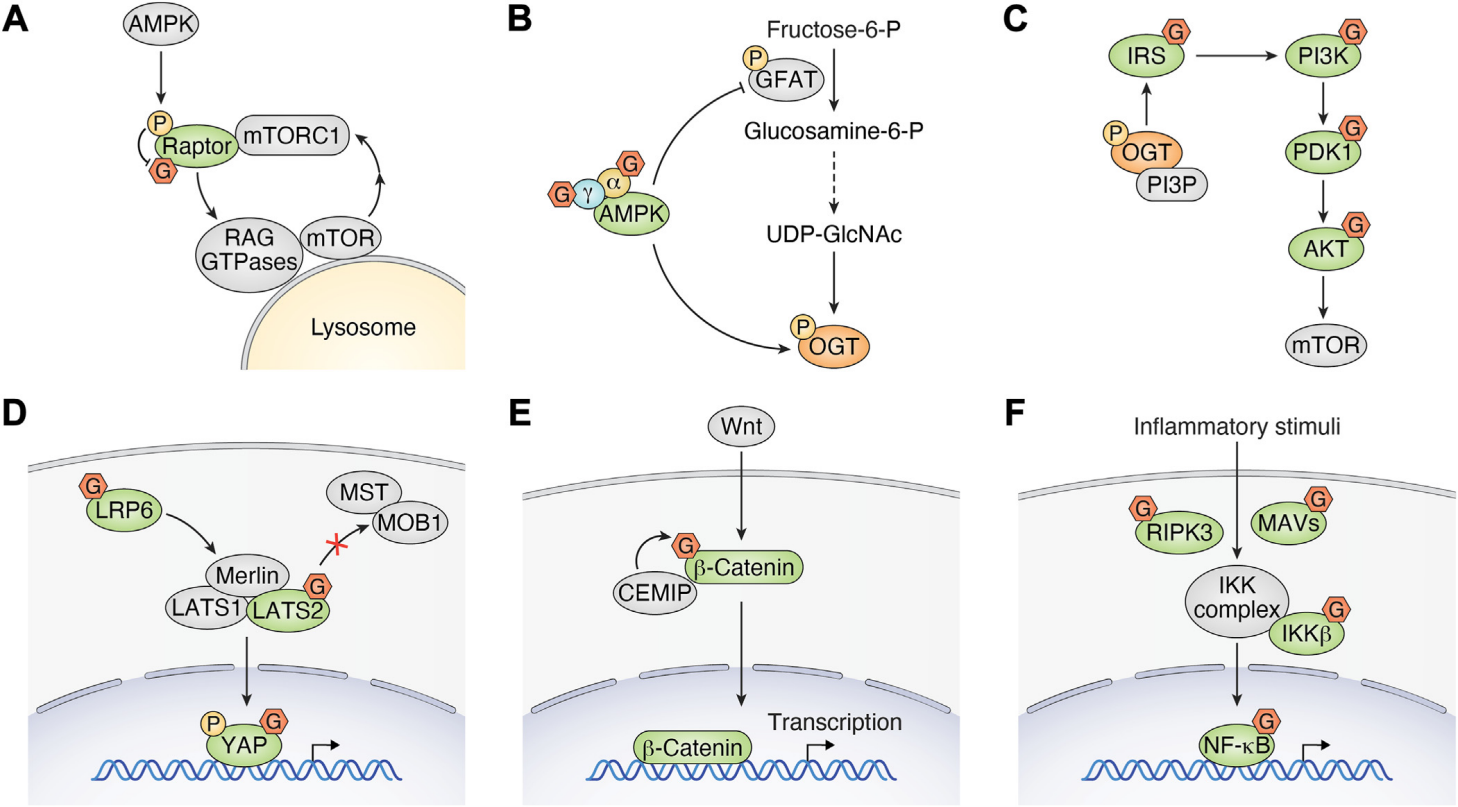
**O-GlcNAcylation regulates various cancer cell signaling pathways**. O-GlcNAcylation occurs on key mediators of multiple cellular signaling pathways, which activates or inactivates these proteins, thus disrupting these signaling pathways to promote tumorigenesis, progression, and metastasis. These signaling pathways include but are not limited to the AMPK‒mTOR pathway (*A*), the AMPK‒HBP‒OGT pathway (*B*), the PI3K‒Akt pathway (*C*), the Hippo‒YAP pathway (*D*), the Wnt pathway (*E*), and the NF‒κB inflammatory pathway (*F*). G: O-GlcNAcylation. P: phosphorylation. HBP, hexosamine biosynthesis pathway; mTOR, mammalian target of rapamycin complex 1; OGT, O-GlcNAc transferase; O-GlcNAcylation, O-linked N-acetylglucosaminylation.

Cell signal transduction is another major effector of protein O-GlcNAcylation (Fig. 4). Upon glucose starvation, the AMPK‒mTOR‒autophagy signaling pathway is activated to reshape cellular metabolism to adapt to nutrient deprivation conditions. O-GlcNAcylation of Raptor at Thr-700 facilitates its interaction with Rag GTPases, thus promoting activation of mammalian target of rapamycin complex 1 (mTORC1) (37). However, a genome-wide CRISPR‒Cas9 screen revealed the major role of OGT in maintaining low mTOR activity and mitochondrial fitness, while OGT loss increases intracellular amino acid levels to promote the lysosomal translocation of mTOR subsequent mTORC1 activation (Fig. 4*A*) (38). This discrepancy implies the complexity of O-GlcNAcylation in cancer, highlighting the possible toxicity of O-GlcNAcylation-targeted therapy in clinical practice. On the other hand, mTOR can regulate protein O-GlcNAcylation in cancer cells through the stabilization of OGT (39, 40). AMPK can also phosphorylate OGT at Thr-444 to inhibit histone O-GlcNAcylation rather than to directly disturb the enzymatic activity of OGT (41, 42). In addition, AMPK phosphorylates glutamine fructose-6-phosphate amidotransferase at Ser-243 to reduce the UDP-GlcNac donor, thus inhibiting O-GlcNAcylation (43). On the other hand, the α and γ subunits of the AMPK kinase complex might also undergo O-GlcNAcylation, which possibly inhibits its kinase activity to impact various downstream pathways (Fig. 4*B*) (41).

Hormones, especially insulin and glucagon, modulate O-GlcNAc signaling to remodel metabolic homeostasis in multiple tissues, such as the liver and fat. In response to insulin, OGT is tyrosine phosphorylated (44) and translocates from the nucleus to the cytoplasm, where it binds to phosphatidylinositol 3,4,5-trisphosphate and catalyzes O-GlcNAcylation of insulin signaling pathway proteins, such as insulin receptor substrate 1/2 (IRS1/2), phosphoinositide 3-kinase (PI3K), and pyruvate dehydrogenase kinase 1 (PDK1), thus suppressing insulin signal transduction (Fig. 4*C*) (44, 45). Upon glucagon stimulation, calcium/calmodulin-dependent kinase II (CaMKII) phosphorylates OGT and promotes the O-GlcNAc modification of ULK1, indicating an essential role for OGT in glucagon-stimulated autophagy, which is supported by the fact that genetic depletion of *OGT* reduces hepatic autophagy (46).

Elevated O-GlcNAcylation causes yes-associated protein (YAP) hyperactivation in cancer cells, and several proteins in the Hippo pathway, such as low density lipoprotein receptor related protein 6 (LRP6), large tumor suppressor kinase 2 (LATS2), and YAP, undergo O-GlcNAcylation, which eventually activates the Hippo pathway. O-GlcNAcylation of LRP6 is essential for its interaction with Merlin, and starvation reduces the O-GlcNAcylation and lysosomal degradation of LRP6, thus allowing Merlin to interact with LATS1/2 and inactivate YAP (47). O-GlcNAcylation of LATS2 occurs at the Thr-436 residue, which disrupts its interaction with MST-MOB1, thus leading to YAP hyperactivation in breast cancer cells (Fig. 4*D*) (48). YAP, the effector of the Hippo pathway that shuttles between the cytoplasm and nucleus in response to LATS-mediated phosphorylation, also undergoes O-GlcNAcylation at Thr-241 (49). This modification leads to YAP stabilization and the activation of the Hippo pathway, which is essential for high-glucose-induced liver tumorigenesis (49). Multiple regulatory proteins in the Hippo pathway are subjected to regulation by O-GlcNAcylation, and these modifications promote the maintenance of the oncogenic role of the Hippo pathway, suggesting the potential of O-GlcNAcylation as an alternative therapeutic choice to target the Hippo pathway in cancer.

β-Catenin, a pivotal factor in the Wnt signaling pathway, is GlcNAcylated at the Thr-41 residue, which protects it from ubiquitination-mediated degradation in colorectal cancer (50). Cell migration-inducing and hyaluronan-binding protein, an adaptor protein of OGT, binds to β-catenin and promotes its O-GlcNAcylation, thus increasing its nuclear translocation and subsequent transcription of glutamine metabolic enzymes, such as glutaminase 1 and glutamine transporters (SLC1A5 and SLC38A2), in colorectal cancer cells (51). These studies highlight the critical role of O-GlcNAcylation in the regulation of the Wnt signaling pathway, which warrants further in-depth studies (Fig. 4*E*).

O-GlcNAcylation also regulates the inflammatory pathway. Viral infection enhances hexosamine biosynthesis pathway (HBP) activity and protein O-GlcNAcylation, and genetic ablation of OGT causes defective antiviral immune responses (52). Mitochondrial antiviral signaling protein, the effector protein in the RNA-sensing antiviral pathway, can be O-GlcNAcylated at Ser-366, which is essential for the K63-linked ubiquitination of mitochondrial antiviral signaling protein and subsequent activation (52). However, another study reported that lipopolysaccharide stimulation leads to attenuated HBP activity and global O-GlcNAcylation levels, and ablation of *OGT* causes innate immune activation and exacerbates septic inflammation, indicating an anti-inflammatory effect of O-GlcNAcylation (8). In lipopolysaccharide-challenged macrophages, receptor-interacting protein kinase 3 (RIPK3) is O-GlcNAcylated at Thr-467, which disrupts its oligomerization and the RIPK3-RIPK1 interaction and thus inhibits necroptosis signaling (8). PDAC has a high HBP flux and O-GlcNAcylation levels, possibly due to high OGT and low OGA expression (53). High glucose levels induce the O-GlcNAcylation of IKK β at Ser-733, thus preventing an inhibitory phosphorylation event at the same site and enhancing NF-κB activity in cancer cells (Fig. 4*F*) (54). The p65 subunit of NF-κB is O-GlcNAcylated at Ser-550 and 551 upon NF-κB activation in PDAC (55), and promotes lung metastasis by activating C-X-C chemokine receptor type 4 (CXCR4) expressions in cervical cancer cells (56).

## O-GlcNAcylation regulates transcription and translation in tumors

**Figure 5:**
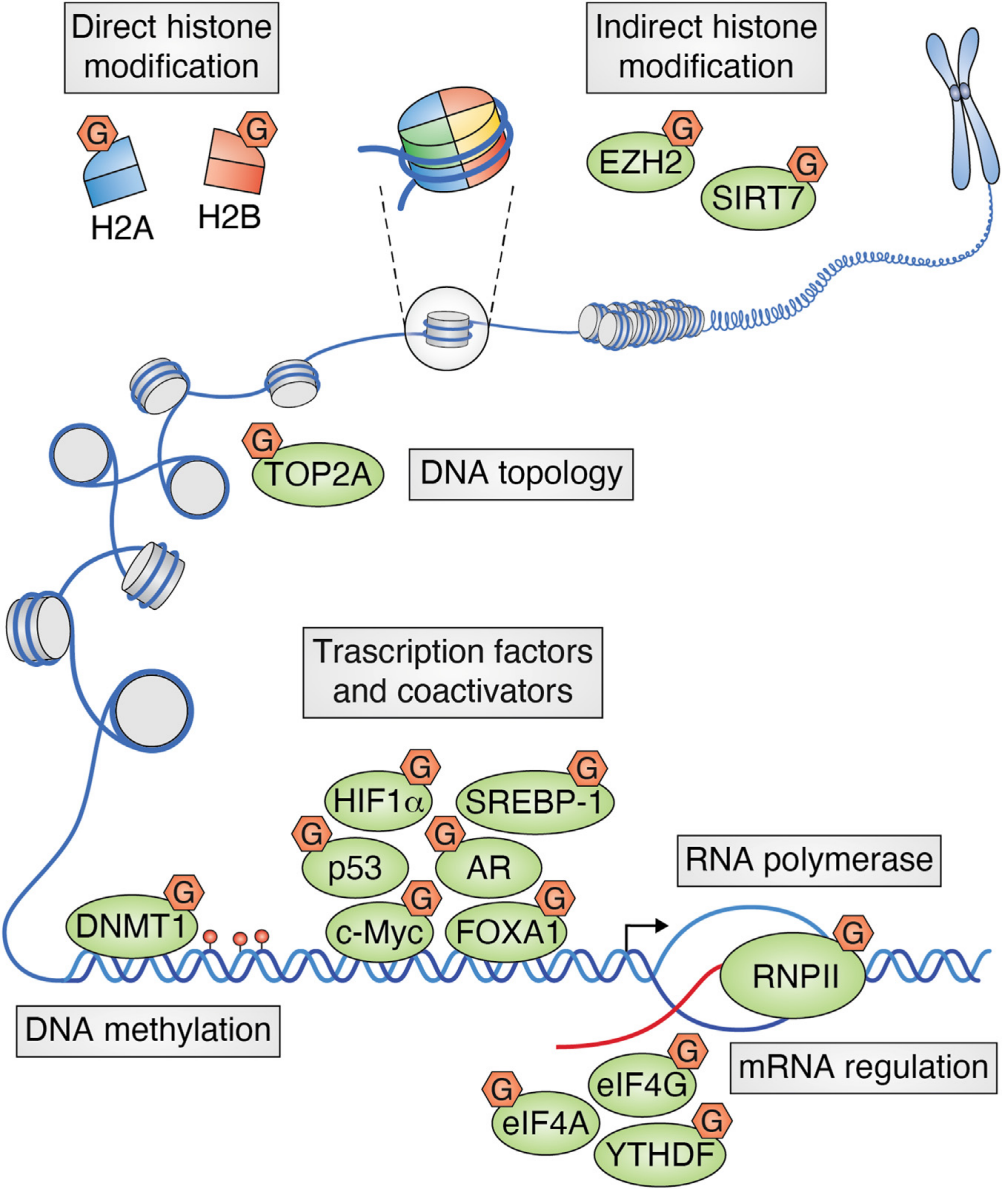
**O-GlcNAcylation regulates transcription and translation in cancer cells.** O-GlcNAcylation occurs on key proteins involved in chromatin organization, transcription mechanisms, and epigenetic regulation, thus reprogramming an oncogenic transcription network to promote cancer. G: O-GlcNAcylation. O-GlcNAcylation, O-linked N-acetylglucosaminylation; PI3K, phosphoinositide 3-kinase.

In addition to the direct modification of metabolic enzymes to remodel cancer cell metabolism, O-GlcNAcylation also occurs on the transcriptional regulatory machinery and epigenetic regulatory proteins, such as histones, transcription factors, and coactivators (Fig. 5). First, O-GlcNAcylation can directly affect DNA methylation by modifying DNA methyltransferase 1 (DNMT1) at its Ser-878 residue, which inhibits the methyltransferase activity of DNMT1 and eventually increases DNA damage and apoptosis (57). Second, histone H2B is GlcNAcylated at Ser-112, providing an anchor for a histone H2B ubiquitin ligase to promote its monoubiquitylation at Lys-120 (58). This GlcNAcylation is widely distributed across chromosomes and colocalizes with Lys-120 monoubiquitination to serve as a transcriptional activation code (58). In addition, GlcNAcylation also occurs on histone H2A at Ser-40 to maintain genome integrity (59, 60). O-GlcNAcylation at Ser-75 of enhancer of zeste homolog 2 (EZH2), which is the enzymatic subunit of the polycomb repressive complex 2 histone methyltransferase, increases its stability and hence facilitates H3K27me3-mediated gene silencing (61). In PDAC, O-GlcNAcylation occurs at Ser-136 of SIRT7, which protects it from degradation, while high expression of SIRT7 leads to hypoacetylation of H3K18 and the subsequent suppression of the transcription of tumor suppressor genes (62). Third, O-GlcNAcylation also regulates DNA topology in part by modifying DNA topoisomerase IIα (TOP2A) at Ser-1469, which enhances its chromatin DNA binding and catalytic activity, leading to resistance to chemotherapy in breast cancer (63). Finally, OGT is recruited to the histone deacetylase complex at promoters, where the transcription corepressor mSin3A targets it to modify and inactivate transcription factors and RNA polymerase II (RNAPII) (64). For example, O-GlcNAcylation of RNAPII on its C-terminal domain controls transcription initiation (65).

The most important transcription factors regulating glycolysis enzymes, such as hypoxia-inducible factor-1α (HIF-1α), p53, and c-Myc, have been reported to be associated with O-GlcNAcylation, all of which promote glycolysis in cancer cells through the transcription of diverse glycolytic enzymes and glucose transporters. Myc is one of the earliest reported substrates of O-GlcNAcylation (66), which occurs on Thr-58, the same site of glycogen synthase kinase-mediated phosphorylation that plays a critical role in mediating its ubiquitination by the F-box/WD repeat-containing protein 7 (FBW7) E3 ligase and subsequent degradation by the 26S proteasome (67). In pancreatic cancer, O-GlcNAcylation of c-Myc not only increases the stability of c-Myc but also promotes the transcription of its downstream genes, such as programmed death-ligand 1 (PD-L1) (68). O-GlcNAcylation of HIF-1α promotes GLUT1 transcription, whereas suppression of O-GlcNAcylation leads to the hydroxylation of HIF-1α and its subsequent proteasomal degradation in human basal-like breast cancers (69). O-GlcNAcylation of p53 at Ser-149 is inversely correlated with its phosphorylation at Thr-155, the COP9 signalosome recognition site of p53. Thus, O-GlcNAcylation of p53 protects it from ubiquitin-dependent proteolysis (70). On the other hand, high glucose levels induce the O-GlcNAcylation of casein kinase II (CK2) and impede CK2-mediated phosphorylation of casein 2, thus releasing Cullin-4 (CRL4) to assemble the CRL4/COP1 E3 ligase complex that degrades p53 (71). Sterol regulatory element-binding transcription factor 1 is the master lipogenic transcription factor, and OGT regulates its protein abundance *via* AMPK (72). O-GlcNAcylation of another transcription factor, SP1, increases sterol regulatory element-binding transcription factor 1 (SREBP1) expression and lipid synthesis in liver and breast cancer cells (73). O-GlcNAcylation at Thr-114 of the TATA-box binding protein blocks its interaction with B-TFIID TATA-box binding protein associated factor 1 (BTAF1) and hence the formation of the B-TFIID complex, leading to gross alterations in lipid storage (74).

Many cancer driver transcription factors also undergo O-GlcNAcylation. In prostate cancer, O-GlcNAcylation occurs on the androgen receptor (AR) protein at the Thr-80 residue next to the Ser-81 phosphorylation site, which leads to stabilization, nuclear translocation, and transactivation of AR (75). Forkhead box protein A1 (FOXA1), a transcription factor involved in epigenetic reprogramming, is also O-GlcNAcylated at the Thr-432, Ser-441, and Ser-443 sites (76). These modifications reshape the interactome of FOXA1 to promote breast cancer metastasis by relocating it onto the chromatin loci of adhesion-related genes and simultaneously recruiting methyl-CpG binding protein 2 (MeCP2) to suppress the transcription of these genes (76). O-GlcNAcylation of the NF-κB subunit p65/RelA at Thr-305 and Ser-319 increases its acetylation at Lys-310 mediated by CREB-binding protein (CBP)/p300, and IKK-mediated phosphorylation at Ser-536, thus increasing its transcriptional activity in PDAC (53, 77).

In addition, O-GlcNAcylation also occurs on protein targets involved in mRNA regulation and protein translation, such as YTHDF1/3. YTHDF proteins are primary readers of N6-methyladenosine (m6A) modifications of mRNAs, which dictate that mRNAs undergo splicing, translation, or degradation. YTHDF1/3, rather than YTHDF2, has a high level of the O-GlcNAc modification, which suppresses YTHDF1/3-mediated translation-promoting functions (78). The translation initiation factors eIF4A and eIF4G are ATP-dependent RNA helicases that are crucial for the assembly of translationally active ribosomes. O-GlcNAcylation of eIF4A at Ser-322/323 disrupts the formation of the translation initiation machinery, suppressing protein synthesis and cell proliferation. On the other hand, O-GlcNAcylation of eIF4G at Ser-61 promotes its interaction with mRNA to induce protein synthesis and cell proliferation, indicating the complexity of translational control (79).

## O-GlcNAcylation in the tumor microenvironment and the immune response

The tumor microenvironment refers to a complex and integrated system formed by the interactions of tumor cells with surrounding tissues, the extracellular matrix, blood vessels, and immune cells that plays a key role in tumor growth, metastasis, and the treatment response. O-GlcNAcylation participates in the regulation of the tumor microenvironment by modifying different substrate proteins. For example, hyaluronidase synthase 2 is a key component regulating the synthesis of hyaluronic acid in the extracellular matrix. O-GlcNAcylation of hyaluronidase synthase 2 at Ser-221 protects it from degradation, whereas a phosphorylation-mimic mutation of the same site reduces its stability, thus regulating hyaluronic acid production (80). O-GlcNAcylation also regulates tumor angiogenesis by modifying the corepressor Sp3, which further inhibits its recruitment to the promoter of angiopoietin-2 (Ang-2), thereby increasing Ang-2 expression and subsequently stimulating neovascularization in tumors (81). In prostate cancer, *OGT* ablation reduces the expression of angiogenic factors, such as vascular endothelial growth factor, matrix metallopeptidase 2 (MMP2), and MMP9, to block angiogenesis (82). In bladder cancer, O-GlcNAcylation promotes angiogenesis largely through the modification of Ser-101 in the seryl-tRNA synthetase (SerRS) protein, which promotes its degradation (83). Recently, immune checkpoints such as PD-L1 have been found to be subjected to O-GlcNAcylation, which protects against lysosomal degradation and promotes tumor immune evasion (84). In addition, O-GlcNAcylation is important for the development, transformation, and differentiation of T cells, whereas *OGT* loss compromises T-cell renewal and clonal expansion (85). T-cell-specific *Ogt*-null mice generated *via* CD4-Cre impair the differentiation and maturity of single-positive CD4+ or CD8+ T cells, although they have similar numbers of double-positive T cells (85). These studies highlight the potential of O-GlcNAcylation-targeted therapy for remodeling the tumor immune microenvironment and urge further in-depth investigations of its synergistic effect with immunotherapy.

## Animal models for the study of O-GlcNAcylation

Various genetically modified mouse models have been developed to gain a more comprehensive understanding of the physiological functions of O-GlcNAc/OGT-related signaling pathways *in vivo*. The *Ogt* mRNA is expressed universally in all mouse tissues, and mice with global *Ogt* knockout (KO) (*Ogt*: X-linked gene, *Ogt*^*-/*^*Y* or *Ogt*^*−/−*^) exhibit embryonic lethality (86). Tamoxifen-inducible whole-body KO of *Ogt* results in lethality in adult mice, which underscores its crucial role in embryonic development and the survival of adult mice (87). UDP-GlcNAc serves as a substrate for OGT and a terminal metabolite of the HBP, which functions as a nutrition sensor. The connection between OGT and HBP has been established using genetically engineered mouse models. Transgenic mice overexpressing *Ogt* in muscle and fat display insulin resistance and elevated leptin levels (87). In contrast, muscle-specific *Ogt* KO in mice results in an increase in energy expenditure, accompanied by increased glucose uptake in both muscle and white adipose tissue (88). In addition, a study involving pancreatic β cell-specific *Ogt* KO mice demonstrated a substantial increase in blood glucose levels 2 months after initiating *Ogt* depletion, which was accompanied by increased β cell apoptosis (87). These findings highlight the critical role of OGT in nutritional sensing and glucose metabolism.

Considering the pivotal roles of the HBP in T-cell development and function, thymocyte-specific *Ogt*-KO mice were examined. The results showed that OGT plays a role in suppressing apoptosis in mature CD4+ and CD8+ single-positive thymocytes (89). Consistent with these findings, thymocyte-conditional *Ogt* KO mice, when bred with thymocyte-specific *Pten* KO mice, which serve as a mouse T-cell acute lymphoblastic leukemia (T-ALL) model, rescued the malignant phenotype of the *Pten*-null mice. This reversed phenotype is likely attributable to the impairment of positive selection in double-positive thymocytes and the suppression of T-cell clonal expansion, both of which are mediated by *Ogt* deletion (85). These findings indicate that OGT enzymatic activity likely plays a critical role in the development of T-cell malignancies, suggesting that OGT may be a potential target for treating T-cell leukemia.

Furthermore, multiple mouse models with conditional *Ogt* deletion have highlighted the essential role of OGT in regulating the immune system. In these models, the depletion of *Ogt* in B cells leads to increased apoptosis of mature B cells and impaired activation of B-cell receptor signaling, underscoring the essential role of OGT in maintaining B-cell homeostasis and immune responses (90). Mice with myeloid-specific *Ogt* deletion exhibit a decrease in cytokine expression induced by influenza A virus infection, highlighting the essential role of OGT in regulating antiviral responses (91). Furthermore, *Ogt* KO in regulatory T (Treg) cells is associated with increased systemic inflammation and the development of an autoimmune phenotype due to impaired Treg effector function (91).

The relationship between OGT enzymatic activity and cancer development was further investigated. To this end, Ser-355 in GLI family zinc finger 2 (GLI2) was found to be a crucial site for O-GlcNAcylation in the promotion of medulloblastoma. Medulloblastoma is a malignancy that affects the cerebellum in children and is caused by sustained Shh (sonic hedgehog) signaling in granule neuron precursors. GLI2, a transcription factor, plays a regulatory role in the sonic hedgehog (Shh) signaling pathway. OGT-mediated GLI2 O-GlcNAcylation impaired the interaction between GLI2 and p300, leading to increased GLI2 transcriptional activity and the promotion of medulloblastoma progression. Deletion of *Ogt* in granule neuron precursor suppressed tumor growth and increased the survival rate of a mouse model of medulloblastoma (92). This study proposed that OGT could be a viable therapeutic target for Shh-activated tumors. Furthermore, the pathological role of O-GlcNAcylation in hepatocellular carcinoma (HCC) has been reported in a ribosomal receptor for activated C-kinase 1 (RACK1) knock-in mouse model. O-GlcNAcylation of RACK1 at Ser-122 is essential for promoting HCC tumorigenesis and chemoresistance. Mechanistically, RACK1 serves as a scaffold protein for multiple signaling components, and its dysregulation is involved in several cancers. In an HCC mouse model, ablation of O-GlcNAcylation at Ser-122 of RACK1 by generating *RACK1*^*S122A/S122A*^ knock-in mice significantly suppressed tumorigenesis, angiogenesis, and metastasis of HCC (93). Collectively, these mouse models provide critical insights into the development of potential therapeutic strategies that target OGT.

The physiological role of OGA *in vivo* has been extensively investigated in genetically engineered mouse models. Like *Ogt*, *Oga* is also widely expressed in mice. *Oga* homozygous whole-body KO leads to perinatal lethality due to delayed embryonic development. KO mice exhibit a smaller body size associated with delayed embryonic development and significantly reduced blood glucose and hepatic glycogen levels (94, 95). Consistent with this finding, the catalytically inactive *Oga* knock-in mouse (*OGA*^*D285A/D285*^^*A*^) displays neonatal lethality. OGA contains an N-terminal OGA catalytic domain and a C-terminal pseudohistone acetyltransferase domain. The lethality observed in knock-in mice suggests that the developmental defect in KO mice is largely attributable to the lack of OGA catalytic activity but is not mediated by the histone acetyltransferase domain (96). Furthermore, hematopoietic stem cell-specific KO of *Oga* in mice impaired the self-renewal of stem cells, diminished progenitor populations, and increased the degree of apoptosis in bone marrow cells. These findings highlight the crucial role of OGA in maintaining the homeostasis of hematopoietic stem cells. Furthermore, in terms of the relationship between OGA and cancer, research has shown that elevated levels of OGT, OGA, and O-GlcNAcylation are observed in colorectal cancer (97). Consistent with this result, heterozygous *Oga* KO in mice suppresses intestinal tumorigenesis on an adenomatous polyposis coli (*Apc*) mutant background and improves the survival rate of the mice (97). In light of the crucial role of O-GlcNAc cycling in cancer, additional *in vivo* analyses are necessary to assess the pathological implications of O-GlcNAcylation in cancer-related and O-GlcNAc cycling signaling pathways regulated by reciprocal OGT/OGA activities.

## Therapeutic approaches to target O-GlcNAcylation in cancer

Multiple small-molecule inhibitors have been developed that either directly inhibit the activity of OGT or OGA or block key enzymes in the HBP to manipulate the O-GlcNAcylation of protein targets (Table 1, see details in other review (98)). These inhibitors include substrate analogs, transition state analogs, glutamine analogs, and others, among which several compounds are in clinical trials, such as Azaserine, DON, and DON precursor DRP-104 (15, 98, 99, 100, 101, 102, 103, 104, 105, 106, 107, 108, 109, 110, 111, 112, 113, 114, 115, 116, 117, 118, 119). Moreover, streptozotocin, a Food and Drug Administration-approved drug for pancreatic neuroendocrine tumors, has also been suggested to be a transition state analog inhibitor of OGA.

**Table 1:** Inhibitors that target the O-GlcNAcylation pathway in cancer

Target	Molecule	Mechanism of action	*In vitro* and *in vivo* effects	Ref.
OGT	Alloxan	Uracil analog of UDP-GlcNAc	*In vitro*: weak inhibition of OGT with off-target effects *In vivo*: ameliorates type 1 diabetes mellitus	(99, 100)
OGT	BADGP	Analog of UDP-GlcNAc	*In vitro*: reduces O-GlcNAc level, but lacks specificity for OGT	(101)
OGT	UDP-5SGlcNAc	5-thiosugar analog of UDP-GlcNAc	*In vitro*: cell-permeable, effective inhibition of OGT	(102)
OGT	5SGlcNAc	Analog of UDP-GlcNAc	*In vitro*: reduces O-GlcNAc level	(102)
OGT	5SGlcNHex	Analog of UDP-GlcNAc	*In vitro*: inhibits OGT *In vivo*: effective at lowering the tissue O-GlcNAc level	(103)
OGT	Compound 2	Analog of UDP-GlcNAc	*In vitro*: cell-permeable, irreversibly inhibits OGT	(104)
OGT	L01		*In vitro*: reduces O-GlcNAc level with low toxicity *In vivo*: effectively inhibits Ogt activity in zebrafish	(105)
OGT	OSMI-1	Hinge interactions with OGT Ala896	*In vitro*: reduces O-GlcNAc level	(106)
OGT	OSMI-3, OSMI-4	Hinge interactions with OGT Ala896	*In vitro*: reduces O-GlcNAc level	(107)
OGA	α-GlcNAc thiolsulfonate	Analog of UDP-GlcNAc	*In vitro*: selectively inhibits short OGA	(108)
OGA	GlcNAcstatins	Analog of GlcNAc	*In vitro*: high potency and selectivity	(109, 110)
OGA	NAG-thiazoline	Transition state analog	*In vitro*: greater selectivity than PUGNAc	(111)
OGA	PUGNAc	Transition state analog, cell-permeable inhibitor	*In vitro*: limited aqueous solubility, low specificity	(112)
OGA	Streptozotocin	Transition state analog	*In vitro*: major off-target effects	(113)
OGA	NButGT	Transition state analog	*In vitro*: less potent, higher selectivity	(114)
OGA	Thiamet G	Derivative of NButGT	*In vitro*: restores O-GlcNAcylation levels with high stability and good water solubility *In vivo*: orally available	(114)
OGA	MK-8719	5-Difluoromethyl derivate of thiamet-G	*In vivo*: increased O-protein levels in PBMCs and brain tissue.	(115)
OGA	Formulaic Ia (LY3372689)		*In vivo*: plasma-concentration-related increase in brain OGA occupancy	(116)
OGA	ASN90		*In vivo*: elevates the O-GlcNAcylation of brain proteins	(117)
HBP	Azaserine	Decreases HBP flux by inhibiting GFAT	*In vitro*: reduces HBP flux, but lacks specificity *In vivo*: in clinical trials	(118)
HBP	6-Diazo-5-oxo-L-norleucine (DON)	Decreases HBP flux by inhibiting GFAT	*In vitro*: reduces HBP flux, but lacks specificity *In vivo*: in clinical trials	(119)

GFAT, glutamine fructose-6-phosphate amidotransferase.

Although O-GlcNAcylation plays an important role in tumors, its physiological function in maintaining the cellular homeostasis of normal tissues is indispensable. Whole-body depletion of *Ogt* or *Oga* leads to a lethal phenotype (86, 94, 95), which undermines the potential toxicity of inhibitors of OGA or OGT that disrupt the universal O-GlcNAcylation of different protein substrates. Therefore, approaches that specifically modulate the O-GlcNAcylation of a tumor-driven target protein are urgently needed for targeted cancer therapy. CIP is an emerging field that has been recently adopted to manipulate protein‒protein interactions (19), especially for modulating specific PTMs of disease-causing proteins, including ubiquitination, phosphorylation, and acetylation. The best known CIP examples are PROTACs, while molecule glues that induce protein‒protein interactions have a longer history, for example, FK506 and rapamycin (20).

**Figure 6:**
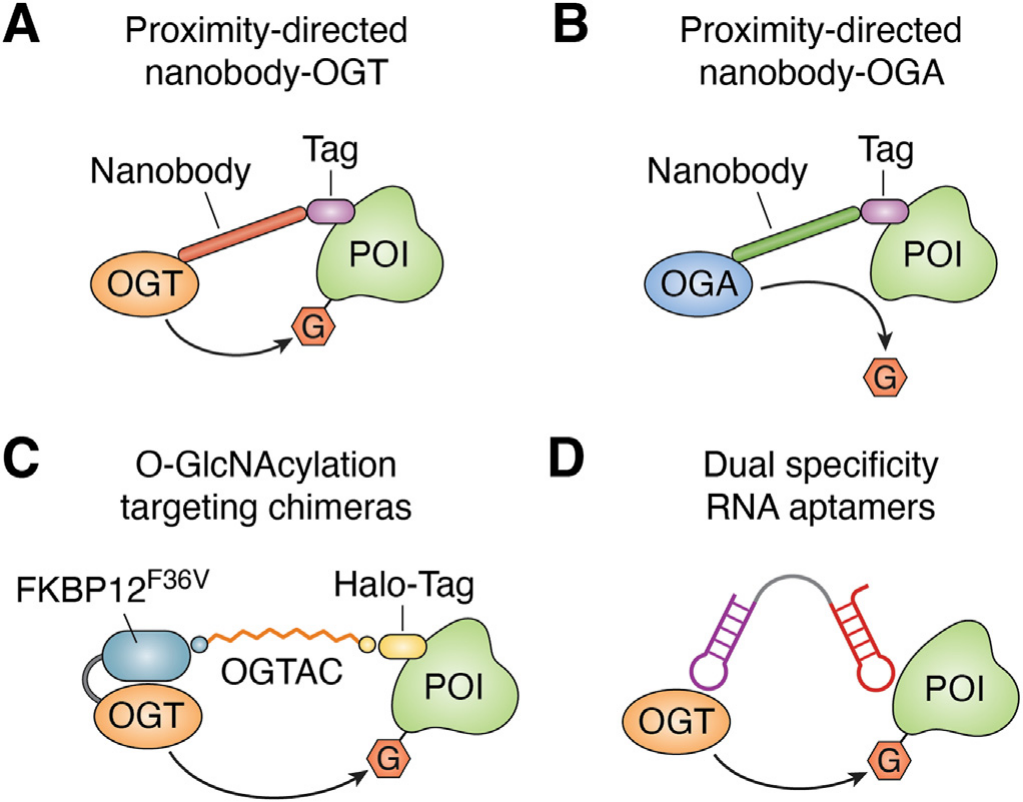
**Chemical-induced proximity method for targeting protein O-GlcNAcylation.***A*, strategy for targeted O-GlcNAc induction using a proximity-directed nanobody-OGT. *B*, design of a nanobody-directed split OGA for selective O-GlcNAc removal from proteins. *C*, concept and target engagement of OGTAC technology. *D*, dual-specific aptamers enhance the O-GlcNAcylation of target proteins. POI: protein of interest. G: O-GlcNAcylation. OGT, O-GlcNAc transferase; O-GlcNAcylation, O-linked N-acetylglucosaminylation; OGTAC, O-GlcNAcylation targeting chimera.

CIPs that modulate the PTM of a protein usually consist of three distinct functional moieties, namely, two ligands that recruit the protein substrate and the PTM enzyme and a linker that connects the two parts (20). Notably, to enable the functional modification of the PTM of the protein substrate, the ligand of the enzyme should bind specifically to the enzyme but not disrupt its enzymatic activity. In addition to small molecules, available ligands for recruiting enzymes in CIP design include DNA/RNA nucleotides and peptides, and ligands of substrate proteins could also be small molecules or antibodies. For example, DNA oligomer-based ligands have been used in PROTAC design to recruit DNA-binding proteins, such as transcription factors and telomeres (120, 121, 122, 123). Due to advances in cyclic peptide and stapled peptide technology, which resolve the stability issue of peptide drugs, peptide-based ligands constitute another class of ligands that can be used in CIP design, and several successful peptide-based PROTAC examples have recently been reported to degrade undruggable targets, such as AR-v7, which escapes the inhibition of canonical AR inhibitors (124). For O-GlcNAcylation, RNA aptamer and nanobody technologies have been adopted to design CIPs (125, 126, 127) (Fig. 6).

The Woo group developed nanobody-based OGT (125) in 2020 and nanobody-based splitOGA methods in 2021 to add or remove O-GlcNAcylation from a specific target protein, respectively (126). In the nanobody-based OGT design, a nanobody of protein of interest (POI) is fused to OGT, thus recruiting OGT to be in proximity and selectively transfer O-GlcNAc onto POIs, such as JunB, c-Jun, Nup62, and α-synuclein (Fig. 6*A*) (125). In the nanobody-based splitOGA design, the OGA protein is split into two parts: a larger part contains the main catalytic domain but has reduced deglycosylase activity without adding a smaller part that is fused with the nanobody. The prototype nanobody-based splitOGA has been proven to be efficient in erasing the O-GlcNAc modification from POIs, such as SP1, c-Jun, c-Fos, JunB, c-Jun, and Nup62 (Fig. 6*B*) (126). Using a tag system, in 2024, the Ng group reported O-GlcNAcylation targeting chimeras (OGTACs), which are bifunctional small molecules that add O-GlcNAcylation to POIs in cells (128). In this design, they use ectopic FKBP12^F36V^-fused OGT as the enzyme and ectopic HaloTag-fused POIs as protein substrates, which illustrates that OGTACs can specifically mediate the O-GlcNAcylation of POIs, including BRD4, CK2α, and EZH2 in cells (Fig. 6*C*) (128). Given that the lack of O-GlcNAcylation mimics the amino acid mutation of the residue for this PTM, previous studies on O-GlcNAcylation have focused mainly on the loss-of-function mutation of serine/threonine-to-alanine (S/T-to-A), which cannot be used to dissect the function of O-GlcNAcylation from other PTMs, such as phosphorylation, on the same residue. Thus, the dual-specificity RNA aptamer provides a powerful tool and paves a new path to study the function of O-GlcNAcylation of specific substrate proteins without affecting other substrates (Fig. 6*D*) (127). Although the relative efficiency and specificity of these chemical engineering methods have been proven, a noticeable drawback is that an ectopically fused enzyme and/or a fused POI needs to be introduced into cells, which is not tolerable in clinical practice, thus restricting their usage only as a research tool in the laboratory.

More recently, using an high-throughput screening method, the Hart group reported the first RNA aptamer that recruits OGT, on the basis of which they linked it to another RNA aptamer of β-catenin, yielding a dual RNA aptamer to bridge endogenous OGT and β-catenin, thus specifically catalyzing the O-GlcNAcylation of β-catenin (127). Furthermore, the aptamer could be developed into an inducible expression system, making it more feasible and controllable in practice (127). Using the dual-specificity RNA aptamer, the biological function of O-GlcNAcylation of β-catenin has been revealed to disrupt the interaction between β-catenin and other proteins and to recruit EZH2 to the gene promoter to remodel the transcriptome (127). Given that OGT and POIs are endogenous proteins and that recent advances in the mRNA vaccine field have provided solutions to resolve the stability and delivery issues of RNA medicine, such dual-specificity RNA aptamer methods might be translated into clinical use in the near future.

## Conclusions

O-GlcNAcylation is a dynamic and reversible PTM of proteins that is involved in almost all cell biological processes and has complex functions in cancer. Many proteins, such as metabolic enzymes, cell signal transduction molecules, and gene expression regulators, are O-GlcNAcylated in response to high glucose or other stimuli to either protect them from degradation or maintain/inhibit their original biological functions in a context-dependent manner. Genetically engineered mouse models, including whole-body or tissue-specific depletion of *Ogt* or *Oga*, reveal the pivotal physiological functions of O-GlcNAcylation in maintaining cellular homeostasis. Small-molecule inhibitors of OGT or OGA have been developed to combat various types of cancers both *in vitro* and *in vivo*. Therefore, drugs that grossly inhibit global O-GlcNAcylation might have serious side effects, thus highlighting the need for alternative approaches to manipulate the O-GlcNAcylation of specific targets accurately without disturbing the functions of other O-GlcNAcylation substrates. To date, several CIPs, including OGTACs and RNA aptamers, have been reported to be able to precisely modulate the O-GlcNAcylation of POIs, suggesting that CIPs might be powerful tools for either studying the biological function of O-GlcNAcylation or serving as therapeutic options in translational studies of human cancers. However, their efficiency and specificity have only been tested in cellular experiments, and further in-depth *in vivo* studies, such as those using genetically engineered mouse model or patient-derived xenograft models, are needed to evaluate their translational potential in the future.

## Conflict of interest

W.W. is a co-founder and consultant for the ReKindle Therapeutics. Other authors declare that they have no conflicts of interest with the contents of this article.
